# When Did the Health Gradient Emerge? Social Class and Adult Mortality in Southern Sweden, 1813–2015

**DOI:** 10.1007/s13524-020-00877-5

**Published:** 2020-05-05

**Authors:** Tommy Bengtsson, Martin Dribe, Jonas Helgertz

**Affiliations:** 1 Centre for Economic Demography, Department of Economic History, Lund University, Lund, Sweden; 2 Institute for Social Research and Data Innovation, Minnesota Population Center, University of Minnesota, Minneapolis, MN 55455 USA

**Keywords:** Adult mortality, Class gradient, Mortality differences, Twentieth century, Sweden

## Abstract

Across today’s developed world, there is a clear mortality gradient by socioeconomic status for all ages. It is often taken for granted that this gradient was as strong—or even stronger—in the past when social transfers were rudimentary and health care systems were less developed. Some studies based on cross-sectional data have supported this view, but others based on longitudinal data found that this was not the case. If there was no gradient in the past, when did it emerge? To answer this question, we examine social class differences in adult mortality for men and women in southern Sweden over a 200-year period, using unique individual-level register data. We find a systematic class gradient in adult mortality emerging at ages 30–59 only after 1950 for women and after 1970 for men, and in subsequent periods also observable for ages 60–89. Given that the mortality gradient emerged when Sweden transitioned into a modern welfare state with substantial social transfers and a universal health care system, this finding points to lifestyle and psychosocial factors as likely determinants.

## Introduction

In most contemporary societies, socioeconomic status is positively associated with health and negatively associated with mortality, with a more accentuated gradient for men than for women (see, e.g., Cutler et al. 2012; de Gelder et al. 2017; Elo 2009; Mackenbach et al. 2003; Smith 1999, 2004). Michael Marmot (2004) called this phenomenon the “Status Syndrome” and maintained that a social gradient in health exists “pretty well everywhere” (p. 16). Likewise, Link and Phelan (1995:81) argued that social conditions are fundamental causes of disease wherever access to resources is unequal. Elo (2009) maintained that although the pathways may vary by context, the resulting health inequalities as such are enduring. Deaton argued, in a similar vein, that “the mortality gradient by income is found wherever and whenever it is sought” (Deaton 2016:1703).

Despite ample empirical evidence demonstrating a socioeconomic health gradient among adults in contemporary developed countries, empirical research on historical contexts is more scarce and less consistent. Some early studies found striking differences in mortality between workers and the upper classes living in cities (Chapin 1924; see also Antonovsky 1967; Deaton 2016), as have some more recent studies (Blum et al. 1990; van Poppel et al. 2009). Others have maintained that mortality differences in the past were small, or even reversed, given that many leading causes of death were highly virulent and mortality primarily depended on exposure—that is, based on residence rather than on nutritional status or access to medical treatment (e.g., Bengtsson and Dribe 2011; Razzell and Spence 2006; Smith 1983; Woods 2004).

Our aim is to explore whether the social gradient in adult mortality has existed in Sweden over the past 200 years and, if not, to identify the point when it emerged. To do so, we study the development of social class differences in adult mortality separately for women and men in a mixed rural and urban area in southern Sweden. Few previous studies have been able to estimate mortality in different social classes using longitudinal individual-level data over such a long period (see, e.g., Razzell and Spence 2006; van Poppel et al. 2009). Finally, we interpret our findings based on a range of possible mechanisms through which social class relates to health and mortality.

The main finding is that the class gradient in adult mortality emerged for women only after 1950 and for men after 1970, first for younger adults and then in the subsequent periods also among older adults. These findings point to factors such as lifestyles and psychosocial stress as likely determinants of the modern health gradient. The fact that our findings for the period after 1970 are consistent with research for Sweden as a whole, as well as other developed countries, makes it likely that the emergence of the adult mortality gradient, as shown in this analysis of southern Sweden, reflects a more general pattern.

## Theory and Previous Research

### Socioeconomic Status and Health

Socioeconomic status is a multifaceted concept that is often measured by education, income, or occupation. A vast body of empirical research on contemporary developed countries has established a strong and robust relationship between each of these measures and health. Higher education is associated with lower adult mortality in Britain (Kunst and Mackenbach 1994; Mackenbach et al. 2003), the United States (Cutler et al. 2012; Halpern-Manners et al. forthcoming; Hayward et al. 2015; Kunst and Mackenbach 1994; Masters et al. 2012), continental Europe (Kunst and Mackenbach 1994), and Scandinavia (Brønnum-Hansen and Baadsgaard 2007; Kravdal 2017; Olausson 1991; Shkolnikov et al. 2012; Steingrímsdóttir et al. 2012; Torssander and Erikson 2010; Vågerö and Norell 1989).

Similarly, occupational rank is related to adult mortality. For example, in the Whitehall studies of British civil servants, a clear health gradient was found in terms of job status (Marmot 2004:27; Marmot et al. 1991). In Sweden, there also appears to be a clear social class gradient in mortality: higher social class is associated with lower mortality overall (Burström et al. 2005b; Hartman and Sjögren 2018; Olausson 1991; Torssander and Erikson 2010; Vågerö and Norell 1989).

There are also differences in mortality by income, wealth, or poverty (e.g., Case and Deaton 2017; Chetty et al. 2016; Elo 2009; Hederos et al. 2018; Smith 1999; Torssander and Erikson 2010). Higher income and/or more wealth is consistently related to lower mortality, even though the strength of the relationship depends on age and is often attenuated when education is controlled for.

Lifestyle factors are often mentioned as important reasons why low socioeconomic status is related to worse health and higher mortality in contemporary settings, through higher smoking prevalence, higher alcohol consumption, greater inactivity, and higher obesity rates (Adler and Stewart 2010; Cavelaars et al. 2000; Elo 2009; Marmot 2004; Norström and Romelsjö 1998; Razzell and Spence 2006; Smith 1999; Vågerö and Norell 1989). Such factors may also indirectly contribute to health given that the success of medical treatment partly depends on lifestyle (Mills et al. 2011).

Differences in access to health care could also be an important explanation for the health gradient. This is especially true in contexts lacking universal provision of health care at a low cost (Adler and Stewart 2010) but also where health care is universal and affordable (van Doorslaer et al. 2000). In the latter case, the health gradient could be related to the underutilization of health services by groups of lower socioeconomic status (Steingrímsdóttir et al. 2012). This could also explain why there is no strong evidence that increased provision of health care actually reduces the health gradient (Smith 1999). Thus, it is not certain that increasing access to affordable health care would eliminate the health gradient in contemporary societies (Adler and Stewart 2010).

Environmental factors could also contribute to the health gradient if different socioeconomic groups systematically were to become exposed to various forms of air and water pollution as well as to other factors influencing the quality of life, such as crime. Adler and Stewart (2010), however, argued that such environmental factors are not of major importance for the modern health gradient.

Marmot (e.g., 2004) stressed the role of psychosocial factors in generating socioeconomic differences in health. Theoretically, *psychosocial factors* refer to the degree to which individuals can control their life situation, focusing in particular on the work situation. Lack of control leads to stress, which negatively affects health through different physiological mechanisms, including increased blood pressure and susceptibility to infection as well as the clogging of blood vessels. What matters is not only *exposure* to stress but also the individual’s ability to *cope* with it. In particular, the combination of high exposure to stress and low levels of coping leads to negative health effects (Adler and Stewart 2010). Typically, low socioeconomic status is associated with both more stress and less ability to cope because of weaker social networks and a lack of other resources (Marmot 2004; Steptoe and Kivimaki 2013). Even though the health penalty linked to low socioeconomic status to a certain extent is context-specific and attenuated in more equal societies, Marmot (2004:27–31) asserted that the links between social position, stress, and health has been present at least since the nineteenth century. Others have argued that the gradient in the Whitehall study is due to health selection into higher classes and not due to stress or the ability to cope with stress (Case and Paxson 2011).

Differences in diet and nutrition are important for socioeconomic differences in mortality. In today’s developed countries, an unhealthy diet is strongly associated with social class (Darmon and Drewnowski 2008), promoting obesity and leading to conditions such as diabetes, hypertension, and heart disease. In the past, malnourished individuals were more likely to die from a number of low-virulent diseases, including measles, diarrhea, tuberculosis, respiratory diseases, pertussis, other intestinal diseases, cholera, leprosy, and herpes (Rotberg and Rabb 1985:305–308). During the first part of the nineteenth century, a pronounced mortality response, particularly among the lower classes, to changes in food prices in Sweden indicates that segments of the population overall were very vulnerable before the onset of modern economic growth (Bengtsson 2004; Bengtsson and Dribe 2005; Bengtsson and Ohlsson 1985). Indeed, improvements in nutritional status have been the leading explanation for the decline of mortality in the nineteenth century (Floud et al. 2011; see also Fogel 2004; McKeown 1976, 1983), but there are also dissenting views (e.g., Easterlin 1996; Livi-Bacci 1991; Szreter 1988).

Finally, it has been suggested that the relationship between socioeconomic status and health in adulthood may have its origin earlier in life (Elo 2009; Smith 1999). Conditions in early life (during the fetal stage and infancy) may have long-lasting impacts on health (e.g., Almond 2006; Barker 1998; Bengtsson and Lindström 2003; Bleakley 2007; Case and Paxson 2008; Elo and Preston 1992; Finch and Crimmins 2004; Quaranta 2013), and exposure to poor nutrition or disease during early life can affect organ development and program the onset of disease in adulthood as well as influence an individual’s cognitive ability. This could in turn affect both health and socioeconomic attainment and thereby explain part of the association between socioeconomic status and health in adulthood that has been observed (e.g., Chandra and Vogl 2010; Cutler et al. 2012).

Although low socioeconomic status is often assumed to have a causal effect on health, some have argued that the direction of causality is more likely to be reversed (e.g., Chandra and Vogl 2010; Cutler et al. 2012; Deaton 2003; Montez and Friedman 2015; O’Donnell et al. 2015; Smith 1999, 2004). Despite this, several empirical studies using quasi-experimental designs have identified a causal effect of socioeconomic status on health or mortality (e.g., Lindahl 2005; Lleras-Muney 2005; Lundborg et al. 2016; Spasojevic 2010).

### A Changing Gradient Over Time?

The socioeconomic mortality gradient in many contemporary settings has been comprehensively mapped, but far less is known about mortality differences by socioeconomic status before the 1970s because of the lack of sufficiently detailed data. Especially glaring is the lack of longitudinal data; most studies for this period are based on cross-sectional data, such as those of Willermé on Paris in 1817, Engels on Manchester in 1850, and Virchow on Upper Silesia in 1847–1848 (see Deaton 2016:1703). In another example, Chapin (1924) used cross-sectional data to examine the mortality differences between taxpayers and nontaxpayers in 1865 in Providence, Rhode Island, indicating the important role of poverty in health and mortality. The findings revealed higher overall mortality as well as mortality by several important causes of death, such as pulmonary tuberculosis, heart disease, and respiratory diseases among the nontaxpayers but only small differences for contagious diseases. In addition, Blum et al. (1990) found substantial socioeconomic differences in remaining life expectancy at age 40 in a study of marriage certificates in Paris in the 1860s, which also included information on age at death of the deceased parents of the bride and groom. A problem found in many of the cross-sectional studies is that the population at risk was not well measured, which results in biased estimates of mortality differences (see Bengtsson and van Poppel 2011).

The ambiguous results from previous research using cross-sectional data for the period before the 1970s have also led to contradictory views in the literature as to whether the socioeconomic gradient in mortality has widened, narrowed, or remained constant since the early phases of the mortality transition. The fact that public health measures, as well as subsidized health care, reached an increasing share of the population during the course of the twentieth century could be expected to have led to a convergence in mortality across social strata (Antonovsky 1967). Because of the redistribution of resources between individuals through the tax system, the level of economic well-being enjoyed by the societies’ poorest people has increased, which should promote a reduction in mortality differences.

This view was challenged by the *fundamental causes theory*, arguing that mortality differences have remained more or less constant over the past 200 years (Link and Phelan 1996; Phelan et al. 2010). Although the specific mechanisms have varied over time, the higher-status groups have always had a mortality advantage because of their greater resources. A recent version of the fundamental causes theory, which is closer to Antonovsky’s view, attempts to take aspects of both the demographic transition and the epidemiological transition theories into account (Clouston et al. 2016). The argument is that as mortality declined, new diseases came to dominate total mortality but with each new disease going through similar phases. Early on, diseases were largely nonpreventable because of a lack of knowledge regarding the causal agents and treatment. In this stage of “natural mortality,” socioeconomic differences in mortality from the disease were usually small, and they could even be in favor of groups with lower socioeconomic status. Social differences arose in the following stage, mainly because of new knowledge on how to prevent disease, which favored the high-status groups, who were quicker to acquire the new information and change their behavior. With a lag, mortality from the disease among the groups with lower socioeconomic status also started to decline; after a while, the rate of improvement was faster among the low-status groups, and inequalities were reduced. This process was repeated disease by disease and in all stages of the mortality transition, except before it began, which is why high-status groups had lower all-cause mortality. In this sense, socioeconomic status is a fundamental cause, even though the precise mechanisms might be different for each disease and in each specific period.

The number of studies using longitudinal data to explore changes in mortality differences by social class over time is low. Van Poppel et al. (2009) found a class gradient in adult mortality in parts of the Netherlands for much of the nineteenth century, which narrowed over time up to 1920, when their study ended. Before then, both the elite and artists lived longer than other groups (van Poppel et al. 2009; van Poppel et al. 2013), but medical professionals did not have this advantage (van Poppel et al. 2016). In fact, medical professionals in Britain also had a shorter life expectancy until approximately 1900 (Woods 2004). British and Russian academics had an advantage in life expectancy at age 50 far back in time, and the gap widened after the 1950s (Andreev et al. 2011). There is evidence of an emerging class gradient in adult mortality in the 1930s in Britain (see also Pamuk 1985; Woods 2000:207).

Recent research on the United States, however, has reported only modest mortality differences by educational attainment for cohorts born during the end of the 1800s and early 1900s (Masters et al. 2012). Additionally, other studies of different historical contexts before the modern period found only minor social differences in adult mortality for men or for both sexes combined (Alfani and Bonetti 2019; Bengtsson and Dribe 2011; Edvinsson 1992; Edvinsson and Broström 2012; Edvinsson and Lindkvist 2011; Smith 1983). Two studies investigating gender differences in mortality by socioeconomic status in Sweden and Estonia found a mortality advantage for higher-status women but not for higher-status men around the turn of the twentieth century (Dribe and Eriksson 2018; Jaadla et al. 2017). In some cases, higher-status men even have a higher mortality than lower-status men, most likely as a result of adverse lifestyles (Dribe and Eriksson 2018; Razzell and Spence 2006).

Other studies using longitudinal data argued that mortality differences in the past were small, or possibly even reversed, because mortality was mainly due to communicable and often highly virulent diseases (e.g., Bengtsson and Dribe 2011; Smith 1983; Woods 2004). Because of the nature of the predominant diseases, the upper classes were possibly even more exposed, which in combination with the lack of effective treatment resulted in higher mortality. Some researchers have also noted that spatial differences in mortality were often much larger than socioeconomic differences in the past (Edvinsson and Lindkvist 2011; Garrett et al. 2001; Reid 1997; Smith 1983; van Poppel et al. 2005; Woods 2000, 2004; Woods et al. 1993).

### Context and Data

We use data from the Scanian Economic Demographic Database (SEDD) for the period 1813–2015. These data consist of individual-level longitudinal information from five rural and semi-urban parishes and, after 1922, the port town of Landskrona in southern Sweden (Bengtsson et al. 2018). The five parishes have a combined population of 4,500 in 1830; 5,500 in 1900; and, together with Landskrona, 37,500 in 2000. The database is one of the very few that can follow individuals across multiple generations from preindustrial times up to the present, with detailed and frequently updated information on occupation and on different demographic outcomes, including migration. The latter is very important because it provides a precise measure of the population at risk. The study population is not a random sample of Sweden but is broadly representative by reflecting conditions shared by populations in similar areas during the time studied (see Bengtsson 2004; Dribe and Helgertz 2016; Dribe et al. 2015; Lazuka 2017:56–60). More specifically, for the period 1813–1920, for which we have data for only the five rural and semi-urban parishes, the area reflects the population density, age, and occupational structure in Sweden outside of Stockholm. In fact, until about 1930, more than half of the Swedish population lived in rural areas. From 1922, the addition of Landskrona allows for the examination of a quintessential industrial town, again reflecting Sweden outside of Stockholm as a whole.

Life expectancy increased throughout the study period. During the twentieth century alone, life expectancy at birth in Sweden rose from 52 to 77 years for men and from 55 to 82 years for women. Mortality rates for men aged 30–34 years fell from 6 to under 1 per thousand between 1900 and 1997 and from 57 to 34 per thousand at ages 70–74 years. Over the same period, mortality for women declined from 6 to 0.4 per thousand at ages 30–34 and from 50 to 19 per thousand at ages 70–74 (Statistics Sweden 1999: table 5.3) As mortality declined, disease patterns also changed, from a predominance of infectious diseases to chronic diseases, such as cardiovascular disease and cancer (Preston 1976). Life expectancy in the study area was very similar to that for Sweden as a whole, although it was slightly higher for cohorts born between 1850 and 1900. Likewise, causes of death followed a pattern similar to that in Sweden as a whole (Lazuka 2017: figs. 6 and 7). The share of mortality due to influenza, pneumonia, and diarrhea fell from more than 30% to 10% in the period 1920–1950. Meanwhile, as in other parts of Sweden, the share of mortality in chronic diseases, cardiovascular diabetes, and cancer increased.

The study period is broken down into six subperiods: 1813–1864, 1865–1919, 1920–1949, 1950–1969, 1970–1989, and 1990–2015. The first period (1813–1864) corresponds to the preindustrial or early industrial phase, when adult mortality was at pretransitional levels and 90% of the population lived in rural areas. For this period in this area, we know that there was a socioeconomic gradient in child and adult mortality between peasants and agricultural laborers in the response to changes in food prices, indicating differences in nutritional status (Bengtsson 2004), which disappeared in subsequent periods (Bengtsson and Dribe 2005). The major health intervention in this period was smallpox vaccination, which started in 1801 and became compulsory in 1816 (Sköld 1996), but there were no pronounced class differences in vaccination rates (Dribe and Nystedt 2003).

Starting in the 1860s, Sweden experienced its industrial breakthrough, with rapid mechanization of agriculture and increasing urbanization and real wages for workers (Schön 2010). From about 1850, adult mortality started its continuous decline (von Hofsten and Lundström 1976). New public health measures, such as improved education for midwives and the establishment of isolation hospitals, were implemented (Lazuka 2018). Investments in improved water and sanitation were also made in urban areas; and in 1900, 50% of the towns had new water systems, and 60% had sewage treatment, both of which contributed to the eradication of the urban mortality penalty by 1930 (Helgertz and Önnerfors 2019). Nevertheless, there were socioeconomic differences in child mortality in this period, particularly in urban areas (Burström et al. 2005a; Edvinsson 1992; Molitoris and Dribe 2016). There were also class differences in marital fertility (Bengtsson and Dribe 2014) as well as strong persistence in socioeconomic status across generations (Dribe et al. 2015), which shows that social class measured by occupation is an important dimension of social stratification in the nineteenth and early twentieth centuries.

In the period 1920–1949, Sweden was in the middle of its industrial transition and showed higher rates of economic growth than most other Western countries (Schön 2010:191) The proportion of men employed in industry in the five rural/semi-urban parishes was 28% in 1930 compared with 32% for the entire country (Statistics Sweden 1936: tables 1 and 2). This was also a period when public welfare institutions were greatly expanded, including pensions, housing allowances, and income compensation during sickness and for work injuries. Nevertheless, the degree of compensation was modest (Edebalk 1996). It was not until 1948, when a new pension system was introduced, that retirees could expect to be able to live on their pension (Edebalk and Olsson 2011). Sulfa, introduced on a large scale in the beginning of the 1940s, instantly reduced pneumonia mortality. The drug was very inexpensive, and there is no evidence of class differences in its use or effects (Lazuka 2020). Regarding lifestyles, most evidence indicates a clear social difference in tobacco use in the period up until the 1940s, with the middle and upper classes smoking more, leading to potentially important health consequences (Dribe and Eriksson 2018; Nordlund 2005).

The subsequent period, 1950–1969, saw rapid economic growth and further development of the Swedish welfare state. In this period, as cigarette smoking grew rapidly, the social differences started to disappear. Then, in the subsequent period, when the adverse health effects of smoking became universally appreciated, the middle and upper classes were the first to stop. This gave rise to the now familiar pattern of smoking being highly correlated with a low socioeconomic status (Nordlund 2005). In this period, smoking was still much more prevalent among men than among women.

The early 1970s, again, saw a continuing expansion of the welfare state, covering almost all aspects of childcare to old-age care, and from income compensation to health care. Meanwhile, levels of education increased, and manual work declined in importance, which implied increased upward social mobility (Dribe et al. 2015).

We use data for the five rural/semi-urban parishes from 1813 to 2015 and for Landskrona from 1922 onward. Information is provided from continuous population registers (a household-based register where information at the individual level is continuously updated), with information on demographic events, including migration to and from households for all individuals in the area. Birth and death registers have been used to add events not recorded in the population registers. Another important characteristic of the data is that migration, into and out of the study area, is comprehensively recorded, meaning that the population at risk is well defined.

From 1968, longitudinal individual-level information covering the entire country is available in administrative registers at Statistics Sweden. Data from these registers have been linked to the historical sample, which has allowed an extension of the database along several dimensions. First, individuals who had ever lived in the study area prior to 1968 but lived elsewhere in the country were followed until 2015 or until death or emigration. Additionally, spouses, parents, grandparents, children, and siblings of individuals belonging to the original population were added to the database if they were alive and living in Sweden sometime after 1967. All individuals added to the sample population were similarly followed until 2015, death, or emigration from Sweden.

The main analysis focuses separately on men and women residing in the study area, of ages 30–89 as well as of ages 30–59 and 60–89 separately. Sensitivity analyses are conducted, comparing different measures of occupation and including only currently married individuals in the sample. In addition, for the two periods after 1970, we compare the sample with a sample excluding foreign-born people and with those people who had ever lived in the area and their relatives regardless of where they lived in Sweden.

We measure social class based on the individual’s and his or her spouse’s occupation for the currently married. Occupational notations have been coded in an internationally comparable coding scheme for historical occupations: the Historical International Standard Classification of Occupations (HISCO) (van Leeuwen et al. 2002). These standardized occupations have subsequently been coded into the Historical International Social Class Scheme (HISCLASS), a 12-category occupational classification scheme based on skill level, degree of supervision, whether manual or nonmanual, and whether urban or rural (van Leeuwen and Maas 2011). In the analysis, we use a six-class version of the scheme, which includes the following classes: higher white-collar workers (HISCLASS 1–2), lower white-collar workers (HISCLASS 3–5), medium-skilled workers (HISCLASS 6–7), lower-skilled workers (HISCLASS 9–10), unskilled workers (HISCLASS 11–12), and farmers (HISCLASS 8). In all analyses, we also include individuals without a registered occupation as a separate category (N/A), which is a very heterogeneous group varying greatly over time. Farmers are also a heterogeneous group, which includes both large-scale farmers who had workers employed at their farms and small-scale farmers who worked on other farms to make their living. This heterogeneity is why it is problematic to fit farmers into the class scheme at any time, a problem exacerbated by the data set encompassing such a long period. Furthermore, the group was already very small by the 1950s.

The remaining five classes, which we focus on, broadly reflect a status hierarchy from the lowest status (unskilled workers) to the highest status (higher white-collar workers). The class scheme is frequently used in historical studies of social stratification and is very similar to other commonly used class schemes in the stratification literature, such as the EGP scheme (see Erikson and Goldthorpe 1992).

For married women, their own social class is unlikely to be a valid indicator of their actual social position given that the share of women with gainful employment was very low well into the twentieth century. Consequently, in the main analysis, we use the highest class within the couple to indicate social class. In the sensitivity analysis, we also show results using individual occupation to measure class.

## Methods

To estimate the association between social class and mortality while controlling for other possible determinants, we estimate a Cox proportional hazards model:

$$ \ln\ {h}_i(a)=\ln\ {h}_0(a)+\boldsymbol{\upbeta} {x}_i, $$

where *h*_*i*_(*a*) is the hazard of death for an individual *i* at duration (age) *a*; *h*_0_(*a*) is the baseline hazard—that is, the hazard function for an individual having the value 0 on all covariates; and **β** is the vector of parameters for the individual covariates (*x*_*i*_). The data set is structured into episodes where individuals are followed from age 30 to 89 and where the episodes are censored when time-varying covariates change value or when an individual moves out of the area.^1^ Models include a full set of control variables: namely, a linear birth year trend, civil status, migration status, and parish of residence. Migration status distinguishes between being born in Sweden or abroad and adjusts for a possible confounding of class associations, which could be especially important after 1950. Because of the very low proportion of foreign-born people before 1920, the variable is not included in the models for the first two periods. Social class is a time-varying covariate in the age group 30–59. For elderly people, we use the highest observed class in the age group 50–59, a time of life when most people have reached their peak in terms of socioeconomic position; occupation records of elderly people could be a rather misleading indicator of social and economic resources because of retirement.

Cox models rely on the assumption of proportional hazards over the duration period. Formal tests (based on the scaled Schoenfeldt residuals, *estat phtest* in STATA) show significant nonproportionality for the missing-occupation category in several periods and for some of the classes in the final period (detailed results not shown). Cumulative hazards plotted over the full duration by class (graphs not shown) reveal nonproportionality mainly at ages over 80 in the final period, where the hazard curves cross. Apart from this, there are no major violations of the proportional hazards assumption, and the model estimates can be interpreted as average differences over the whole duration. In the sensitivity analysis, we also separately present results for two age groups to ensure that our results and interpretations are not affected by the nonproportionality at higher ages. As a sensitivity analysis, we also estimated a Cox model stratified by parish of residence instead of including it as a control variable. The model yielded practically identical results (not shown).

### Descriptive Statistics

Table 1 displays the descriptive statistics of the main sample, containing the five rural/semi-urban parishes during the entire period, from 1813 to 2015, with the industrial port town of Landskrona included from 1922 onward. Class, defined as the highest status in the household among married couples, shows quite dramatic changes, which are explained by fundamental structural changes taking place as Sweden transformed from a poor agricultural society into a rich welfare state. In the first period, 1813–1864, before the industrial breakthrough, approximately one-third of working-age men were farmers, and approximately 20% of unskilled workers mainly worked on farms. Approximately 2% belonged to the class of higher managers and professionals. In total, this implies that approximately 5% worked in white-collar occupations, and 91% worked in manual occupations, leaving 4% without a classified occupation. The distribution for women is very similar but with a higher proportion not having a registered occupation. Unmarried women without an occupation account for this higher proportion having missing occupation.

**Table 1:** Descriptive statistics of the main study sample: The five rural/semi-urban parishes (1813–2015) and Landskrona (1922–2015)

	1813–1864	1865–1919	1920–1949	1950–1969	1970–1989	1990–2015
A. Men						
Family class (%)						
Higher white-collar	1.5	2.5	6.5	10.1	8.4	11.0
Lower white-collar	3.1	8.2	19.9	30.3	38.3	36.4
Medium-skilled	4.8	12.0	25.9	25.7	19.6	13.4
Lower-skilled	35.6	30.6	24.1	22.8	21.8	18.9
Unskilled	20.7	21.6	16.2	6.7	3.0	3.0
Farmers	30.3	20.8	5.7	2.9	2.9	1.4
NA	4.0	4.2	1.8	1.3	6.0	15.9
Individual class (%)						
Higher white-collar	1.5	2.4	6.4	9.4	7.2	9.2
Lower white-collar	2.9	7.8	17.9	23.1	29.7	29.1
Medium-skilled	4.8	11.9	25.0	28.0	22.1	15.9
Lower-skilled	30.2	24.4	23.3	24.8	26.0	21.5
Unskilled	26.2	28.0	19.6	9.8	4.1	3.7
Farmers	30.4	21.1	5.9	3.2	3.3	1.8
NA	4.1	4.4	1.9	1.7	7.6	18.8
Year of birth (mean)	1797	1843	1889	1909	1927	1949
Civil status (%)						
Never married	14.5	16.8	21.2	16.0	14.4	23.6
Currently married	74.7	72.9	73.1	77.4	72.9	57.8
Previously married	10.8	10.2	5.7	6.6	12.7	18.6
Migrant status (%)						
Swedish-born	99.6	98.4	96.8	92.8	81.0	75.6
Foreign-born	0.4	1.6	3.2	7.2	19.0	24.4
Parish (%)						
Hög	9.9	9.8	1.8	1.1	0.6	0.3
Kävlinge	10.8	22.7	11.6	10.7	10.7	14.4
Halmstad	17.3	15.1	2.0	0.8	0.5	0.2
Sireköpinge	18.1	23.3	4.5	2.5	1.7	1.0
Kågeröd	44.0	29.2	8.0	5.5	4.4	5.1
Landskrona	––	––	72.2	79.4	82.1	79.1
Person-years	34,225	61,744	179,837	189,241	247,332	390,866
Number of deaths	799	1,162	2,203	2,861	4,435	6,261
B. Women						
Family class (%)						
Higher white-collar	0.8	2.0	5.5	8.9	7.6	9.4
Lower white-collar	1.9	6.5	20.5	30.5	38.7	39.0
Medium-skilled	3.6	9.7	20.9	20.9	15.7	8.9
Lower-skilled	40.7	38.5	25.4	23.2	20.9	20.3
Unskilled	14.5	13.4	9.5	5.0	4.6	5.3
Farmers	21.9	14.5	3.9	2.1	2.2	1.2
NA	16.6	15.4	14.2	9.4	10.4	15.9
Individual class (%)						
Higher white-collar	0.0	0.5	1.5	2.2	2.8	4.5
Lower white-collar	0.1	1.9	10.8	20.7	28.6	34.7
Medium-skilled	0.4	2.0	6.9	7.4	7.4	5.0
Lower-skilled	29.8	35.4	21.4	28.0	24.0	25.1
Unskilled	11.0	3.7	4.1	9.3	10.0	8.4
Farmers	0.0	0.2	0.1	0.2	0.7	0.8
NA	58.7	56.3	55.3	32.3	26.4	21.5
Year of birth (mean)	1797	1843	1888	1908	1925	1947
Civil status (%)						
Never married	16.9	18.2	28.0	17.2	9.3	15.5
Currently married	66.4	65.0	63.3	69.1	64.9	53.1
Previously married	16.7	16.8	8.6	13.7	25.7	31.4
Migrant status (%)						
Swedish-born	99.8	98.9	97.0	92.9	77.9	72.2
Foreign-born	0.2	1.1	3.0	7.1	22.1	27.8
Parish (%)						
Hög	9.4	9.6	1.6	0.9	0.5	0.2
Kävlinge	10.7	22.2	11.5	10.5	10.7	14.8
Halmstad	17.0	15.3	1.9	0.7	0.4	0.2
Sireköpinge	17.4	24.2	4.3	2.2	1.5	0.8
Kågeröd	45.5	28.7	6.6	4.8	4.0	4.7
Landskrona	––	––	74.1	80.8	83.0	79.3
Person-years	35,678	65,623	192,859	198,899	262,650	410,594
Number of deaths	815	1,262	2,145	2,583	3,364	5,409

*Source:* The Scanian Economic Demographic Database (Bengtsson et al. 2018).

In the following period, after the industrial breakthrough, the proportion of lower-skilled workers declined, and the medium-skilled and lower-white collar increased. In the period 1920–1949, when Landskrona is included in the sample, white-collar classes reached more than 25% to approach 50% after 1970. Clearly, most of the increase in the white-collar group is due to an increase in the group of lower white-collar workers. In contrast, among the blue-collar workers, there was a dramatic decrease among lower-skilled and unskilled workers; instead, there was an increased proportion of skilled workers. Naturally, the proportion of farmers also decreased a great deal, particularly when Landskrona was included in 1922. The development over time is very similar for women and men, as long as we look at family class.

When we measure class at the individual level instead of using the highest class in the family, the distributions are highly similar for men but somewhat different for women, as could be expected. In particular, when individual class instead of family class is used for women, the proportion of farmers is much lower, and the proportion without an occupation is much higher. Within each sex, the group of individuals without a reported occupation is heterogeneous over time. The consistently higher share among women in the periods before 1970 is due to their considerably lower labor force participation. In the two final periods, the proportion without a reported occupation increases substantially to approximately 16%. This could be partly related to the dramatic increase in the number of 45- to 60-year-olds receiving disability pensions in Sweden from the 1970s onward, with a peak in the early 1990s, when approximately 20% of all 55- to 59-year-olds received disability insurance and hence did not work (Jönsson et al. 2012). Another reason for the high proportion of missing occupation in the final periods is that occupation in the period 1970–2001 was reported less frequently than before and after. Consequently, individuals moving into the study area or transitioning to the labor force between two points in time when occupation was reported will be observed with their occupation only at the latter point in time. Similarly, the low proportion of individuals moving into and out of the study area between the two times of reporting will never have a recorded occupation.

Approximately 75% of men in the first period were currently married, and this figure remains relatively constant until the final period, when it declines to just below 60%. The trend for women is similar, but the proportions of people who were currently married are lower, with the proportion of people who were previously married being higher, as could be expected from gender differences in life expectancy.

The proportion foreign-born increased dramatically over time, from less than 1% in the first half of the nineteenth century to approximately 25% in the final period. This reflects the transition of Sweden from a country of emigration to a country of immigration, especially in the period after 1950. Last, there are pronounced differences over time in the distribution of the population across the parishes covered by the data set. The population residing in parishes that remained largely rural throughout the period (Hög, Halmstad, Kågeröd, and Sireköpinge) declined dramatically, but the population of Kävlinge increased as it transformed from a rural village to a semi-urban municipality. After 1922, the industrial city of Landskrona dominates the study population.

### Main Results

Table 2 shows estimates of the association between social class and adult mortality from separate models for each period, using the main sample of the people living in the study area. In these models, social class is based on the highest class in the family for those people who are married and the individual’s own class for the single people.

**Table 2:** Cox proportional hazards estimates of mortality at 30–89 years by social class in the main study sample: The five rural/semi-urban parishes (1813–2015) with Landskrona (1922–2015)

	1813–1864	1865–1919	1920–1949	1950–1969	1970–1989	1990–2015
Men						
Higher white-collar	1.254	0.969	1.005	1.027	0.906	0.760**
Lower white-collar	1.064	0.873	1.083	0.948	0.989	0.830**
Medium-skilled	1.062	0.829^†^	0.994	0.892*	1.065	0.913*
Lower-skilled	1	1	1	1	1	1
Unskilled	1.630**	1.041	0.942	0.927	1.255**	1.181*
Farmers	0.978	0.933	1.190^†^	0.868	0.838^†^	0.807**
NA	1.296	0.809	0.946	0.974	1.951**	1.870**
Women						
Higher white-collar	1.040	0.544^†^	1.010	0.736**	0.736**	0.705**
Lower white-collar	0.999	0.760	0.945	0.902	0.846**	0.859**
Medium-skilled	1.174	0.771^†^	0.954	0.892^†^	0.941	1.097^†^
Lower-skilled	1	1	1	1	1	1
Unskilled	1.344**	0.999	1.025	1.026	0.970	1.097
Farmers	1.088	0.859	1.019	1.093	0.985	0.889
NA	1.225^†^	1.098	1.073	1.265**	1.119*	1.285**

*Notes:* All models control for year of birth, migration status (not included for the first two periods), civil status, and parish of residence. The number of events and time at risk are given in Table 1. All models are statistically significant below the 5 % level based on a chi-square test. Social class is measured by the highest social class in the family for married people.

*Source:* See Table 1.

^†^*p* < .10; *p < .05; **p < .001

The final two periods, 1970–1989 and 1990–2015, show a clear class gradient for men, going from higher white-collar workers with the lowest mortality to the unskilled workers with the highest mortality. Unskilled men had 39% higher mortality than higher-white collar workers in 1970–1989 and 55% higher mortality in the final period. For women, there is also a clear gradient of a similar magnitude, with unskilled workers having 32% and 56% higher mortality, respectively, than higher white-collar workers.

In the period 1950–1969, there is no class gradient in mortality among men; if anything, higher classes had a higher mortality even if the difference is minor. However, during this period, the gradient for women is very similar to the following period, with 39% higher mortality for unskilled workers than white-collar workers. Hence, the social gradient emerged earlier for women than for men and became stronger for both sexes in the final period.

For the period 1920–1949, we find an association between social class and mortality outside the farmer group neither for men nor for women. The hazard ratios are all close to 1 and are not statistically significant. Prior to 1920, there are some statistically significant mortality differences by class for men, but there is not a social gradient in the standard sense (the lower the class, the higher the mortality). Instead, the mortality pattern among men in the first period (1813–1864) is U-shaped, with higher mortality for higher white-collar and unskilled workers. Unskilled workers had 63% higher mortality than low-skilled workers. Although the coefficient is not significant, higher white-collar workers had 25% higher mortality than low-skilled workers, a pattern that vanishes in the following period. Likewise, there are some class differences in adult mortality for women prior to 1920 but not a consistent class gradient. In the 1813–1864 period, unskilled women had much higher mortality than low-skilled women, but the difference is not as large as for men. In the 1865–1919 period, the pattern is more unclear, with lower mortality for higher classes, albeit barely statistically significant.

Tables 3 and 4, respectively, show separate models for the age groups 30–59 and 60–89. Overall, the patterns are similar in the two age groups for both men and women with regard to the emergence of the class gradient. In addition, the patterns in the earlier periods are similar, with higher mortality among the unskilled in the first period. However, the class gradient, and especially the advantage of the higher classes, appears later in the older cohorts than in the younger cohorts.

**Table 3:** Cox proportional hazards estimates of mortality at 30–59 years by social class in the main study sample: The five rural/semi-urban parishes (1813–2015) with Landskrona (1922–2015)

	1813–1864	1865–1919	1920–1949	1950–1969	1970–1989	1990–2015
Men						
Higher white-collar	1.389	0.856	0.819	0.940	0.695*	0.795
Lower white-collar	0.876	0.735	1.120	0.992	0.742**	0.837
Medium-skilled	1.015	0.850	0.973	0.874	0.974	0.789
Lower-skilled	1	1	1	1	1	1
Unskilled	1.500**	1.070	0.977	1.044	1.067	1.360
Farmers	0.873	0.928	0.828	0.992	0.598	0.382
NA	1.596^†^	1.261	1.601*	1.834*	3.645**	3.251**
Women						
Higher white-collar	1.260	0.637	0.872	0.597*	0.674	0.524**
Lower white-collar	0.905	0.845	0.797*	0.804^†^	0.792	0.608**
Medium-skilled	1.303	0.724	0.830^†^	0.925	1.094	0.679^†^
Lower-skilled	1	1	1	1	1	1
Unskilled	1.444*	0.962	1.079	1.041	1.063	0.786
Farmers	1.112	0.964	0.962	0.552	0.708	1.187
NA	1.487*	1.306	1.218	2.691**	2.985**	3.300**

*Notes:* All models control for year of birth, migration status (not included for the first two periods), civil status, and parish of residence. The number of events and time at risk are given in Table 1. All models are statistically significant below the 5 % level based on a chi-square test. Social class is measured by the highest social class in the family for married people.

*Source:* See Table 1.

^†^*p* < .10; *p < .05; **p < .001

**Table 4:** Cox proportional hazards estimates of mortality at 60–89 years by social class in the main study sample: The five rural/semi-urban parishes (1813–2015) with Landskrona (1922–2015)

	1813–1864	1865–1919	1920–1949	1950–1969	1970–1989	1990–2015
Men						
Higher white-collar	0.979	1.011	1.157	1.080	0.935	0.759**
Lower white-collar	1.323	0.987	1.050	0.953	1.048	0.827**
Medium-skilled	1.184	0.812	1.022	0.902^†^	1.077	0.919^†^
Lower-skilled	1	1	1	1	1	1
Unskilled	1.684**	1.040	0.910	0.897	1.304**	1.163^†^
Farmers	1.093	0.929	1.297*	0.839	0.848^†^	0.802**
NA	1.163	0.639*	0.870	0.857	1.229*	1.353**
Women						
Higher white-collar	0.654	0.488	1.191	0.812	0.750**	0.745**
Lower white-collar	0.969	0.668	1.106	0.949	0.870**	0.891**
Medium-skilled	0.800	0.837	1.090	0.881	0.921	1.120*
Lower-skilled	1	1	1	1	1	1
Unskilled	1.137	1.003	0.969	1.014	0.959	1.105^†^
Farmers	1.036	0.785^†^	1.057	1.251	1.041	0.878
NA	0.996	1.046	1.074	1.199**	1.033	1.140**

*Notes:* All models control for year of birth, migration status (not included for the first two periods), civil status, and parish of residence. The number of events and time at risk are given in Table 1. All models are statistically significant below the 5 % level based on a chi-square test. Social class is measured by the highest social class in the family for married people.

*Source:* See Table 1.

^†^*p* < .10; *p < .05; **p < .001

### Sensitivity Analysis

The main results are based on family social class (highest class in family for the currently married) of the population residing in the study area. In this section, we test the robustness of the main findings using a different study population, using different measures of class, and excluding nonmarried and foreign-born people. All results are presented in Tables 5 and 6.

**Table 5:** Sensitivity analysis related to measure of social class and marital status: Hazard ratios of mortality from Cox proportional hazards models

	1813–1864	1865–1919	1920–1949	1950–1969	1970–1989	1990–2015
A. Social Class Based on Own Occupation in the Main Study Sample						
Men						
Higher white-collar	1.328	0.982	1.033	1.038	0.895^†^	0.784**
Lower white-collar	1.160	0.931	1.126^†^	0.954	0.956	0.861**
Medium-skilled	1.131	0.817^†^	1.026	0.871**	1.021	0.944
Lower-skilled	1	1	1	1	1	1
Unskilled	1.708**	1.023	0.968	0.937	1.105	1.071
Farmers	1.034	0.917	1.211^†^	0.871	0.822*	0.785**
NA	1.435*	0.832	0.946	0.994	1.866**	1.839**
Women						
Higher white-collar	––^a^	0.425	0.826	0.851	0.673**	0.799*
Lower white-collar	––^a^	0.702	0.853	0.944	0.834**	0.893**
Medium-skilled	––^a^	0.654	0.869	1.038	1.006	1.226**
Lower-skilled	––^a^	1	1	1	1	1
Unskilled	––^a^	0.846	1.160	1.073	1.015	1.160**
Farmers	––^a^	0.779	0.839	0.500	0.691	0.904
NA	––^a^	0.991	1.102	1.223**	1.192**	1.425**
B. Only Currently Married People Aged 30–89 in the Main Study Sample						
Men						
Higher white-collar	1.051	0.770	0.951	1.050	0.984	0.744**
Lower white-collar	0.845	0.895	1.039	0.989	1.040	0.825**
Medium-skilled	1.036	0.796	1.027	0.919	1.156*	0.905
Lower-skilled	1	1	1	1	1	1
Unskilled	1.411*	1.261^†^	0.942	0.944	1.577**	1.765**
Farmers	0.915	0.940	1.011	0.760^†^	0.768*	0.763**
NA	1.133	0.676	1.025	1.104	1.738**	1.982**
Women						
Higher white-collar	0.978	0.643	1.118	0.712*	0.742*	0.551**
Lower white-collar	0.932	0.766	1.001	0.849^†^	0.857^†^	0.717**
Medium-skilled	1.083	0.797	1.033	0.775**	0.882	0.794**
Lower-skilled	1	1	1	1	1	1
Unskilled	1.150	1.117	0.959	0.926	0.705	1.099
Farmers	1.035	0.880	0.913	1.182	0.913	0.744*
NA	1.215	1.035	1.033	2.739**	0.857	1.098

*Notes:* All models control for year of birth, migration status (not included for the first two periods), civil status, and parish of residence. The number of deaths and person-years at risk are given in Table 1. All models are statistically significant below the 5 % level based on a chi-square test. For models in panel B, social class is measured by the highest social class in the family for married people.

*Source:* See Table 1.

^a^Not estimated because of the few cases in the white-collar, medium-skilled, and farmer groups.

^†^*p* < .10; *p < .05; **p < .001

**Table 6:** Sensitivity analysis related to immigration and place of residence: Hazard ratios of mortality from Cox proportional hazards models for 1970–2015

	Men		Women	
	1970–1989	1990–2015	1970–1989	1990–2015
A. Main Sample, Excluding Foreign-born People				
Higher white-collar	0.928	0.849**	0.729**	0.731**
Lower white-collar	1.019	0.943	0.866**	0.929^†^
Medium-skilled	1.062	0.966	0.922	1.066
Lower-skilled	1	1	1	1
Unskilled	1.242**	1.149	0.966	1.062
Farmers	0.851^†^	0.887	1.008	0.971
NA	1.927**	1.969**	1.085	1.217**
B. Whole Population, Regardless of Location in Sweden				
Higher white-collar	0.856**	0.736**	0.799**	0.694**
Lower white-collar	0.954*	0.865**	0.844**	0.848**
Medium-skilled	1.030	0.961*	1.008	1.073**
Lower-skilled	1	1	1	1
Unskilled	1.216**	1.031	1.055	1.113**
Farmers	0.739**	0.778**	0.868*	0.926*
NA	1.740**	1.949**	1.235**	1.265**

*Notes:* All models control for year of birth, migration status (not included for the first two periods), civil status, and parish of residence. The number of deaths and person-years at risk are given in Table 1. All models are statistically significant below the 5 % level based on a chi-square test. Social class is measured by the highest social class in the family for married people.

*Source:* See Table 1.

^†^*p* < .10; *p < .05; **p < .001

In panel A of Table 5, we define social class based on the individual’s occupation and class rather than for the couple. For men, the results are highly similar to the main results and confirm the emergence of class differences in mortality from about 1970. As expected, it does not matter a great deal for men whether we measure social class for individuals or couples. For women, the pattern is quite similar to the one for class measured at the family level, despite some differences. The lower mortality for higher white-collar women before 1970 is no longer statistically significant when looking at their own class, making it more similar to men. However, women’s own class is a problematic measure, as shown by the high proportion of women with no occupation (see Table 1). At least until the late 1960s, the social class of their husbands is more important for the living conditions of wives than their own social class. This is a period when Sweden largely conformed to a male breadwinner model, and it was actually not until the period after 1970 that the dual-earner household became the norm (Stanfors and Goldscheider 2017). Looking only at the currently married population, in panel B of Table 5, the overall pattern is highly similar to the main results.

In panel A of Table 6, we exclude the foreign-born people from the post-1970 period, when Sweden had been transformed from an emigration country to an immigration country, which especially affected Landskrona. As is clear from Table 1, 20% to 28% of the men and women in the sample in this period were foreign-born, and it is possible that their class structure and mortality levels differed from natives and affected the overall patterns. However, this is not the case: the results in the two samples are highly similar.

Finally, in panel B of Table 6, we look at the population residing all over Sweden and at people who either originated in the study area or whose parents or grandparents were observed there. This population can be followed in the national registers only after 1967, and hence we can include only the two final periods. Naturally, the population is much larger when including people from the entire country. The total time at risk in the two periods is more than 12 million person years compared with 1.4 million in the main sample used in Table 2. (The number of deaths is approximately 120,000 compared with 19,000 in the main sample.) The first thing to note is the close similarity in the patterns for this population and our main analysis in Table 2. This is reassuring because it confirms that the sample including the rural areas and Landskrona is representative of the country as a whole. It is also important to note that we can reproduce the mortality gradient in the final period that has been shown in other studies using similar data but with somewhat different selection criteria, class schemes, and variable definitions. In fact, our main results for ages 30–59 in the final period are very similar to those of a study of the entire country, examining individuals at ages 35–59 years in the period 1990–2003 (Torssander and Erikson 2010). According to this study, unskilled men had 87% higher mortality than higher professionals, and unskilled women had 36% higher mortality. The corresponding figures in our analysis of the last period, which ends in 2015 and not 2003, are 71% and 50%, respectively (Table 2).

Overall, the sensitivity analysis shows that our main findings of the late emergence of the mortality gradient are robust to changes in measurement of social class as well as to the possible impact of immigration and marital status. Expanding the sample to include people who migrated out of the area but still live in Sweden produces similar results. Moreover, our findings are corroborated by the similarity between the mortality gradient found in our regional study population and that of the country as a whole during the periods when they overlap—that is, 1880–1920 (Dribe and Eriksson 2018) and 1990–2003 (Torssander and Erikson 2010)—and are also highly similar to many other Western countries in the post-1970 period.

## Conclusion

Our study of southern Sweden shows that contrary to the established view, the health gradient is quite a recent phenomenon. The class gradient in mortality at ages 30–59 emerged only after 1950 for women and after 1970 for men, appearing even later at ages 60–89. Our estimates for 1990–2015 are close to what has been found for the entire country (Torssander and Erikson 2010). Before it emerged, in the period 1920–1949 for women and 1920–1969 for men, the mortality differences were very small and not statistically significant. Going back to the first part of the nineteenth century, we find that unskilled men and women had a higher mortality than low-skilled workers in the countryside. For men, we also find that higher classes had higher mortality than low-skilled workers in this period. This creates a U-shaped relationship between social class and mortality, similar to what is found for the entire country (Dribe and Eriksson 2018). The higher mortality among the unskilled people in this period is consistent with previous studies showing that the working class was vulnerable to short-term variation in food prices in the period up to 1865 but not afterward, which is indicative of a nutritional vulnerability that the higher classes did not experience (Bengtsson 2004; Bengtsson and Dribe 2005).

Given that our findings for the period after 1970 are in line with research for both Sweden as a whole and other developed countries, the historical emergence of the mortality gradient, as pictured here for southern Sweden, is likely part of a more general pattern. The question is why the social gradient in adult mortality emerged only in the second half of the twentieth century, when Sweden developed into a modern welfare state with general health care provision, pension systems that one could live on, housing allowances, and income loss compensation.

The fact that the class gradient emerged earlier for women than for men makes it unlikely that health care was a key factor given that we would expect it to affect both sexes in a similar way. Instead, lifestyle factors—such as smoking, alcohol consumption, diet, and exercise—have become strongly associated with social class. Indeed, differences in lifestyles between classes are likely one important reason for the delayed emergence of the mortality gradient among men (and to a lesser extent among women) at ages 60–89. Individuals in the older age group in the period 1970–1989 had similar smoking histories regardless of social class, while younger adults show clear class differences (Nordlund 2005).

Psychosocial stress related to modern working life and social position is another factor that is likely to contribute to the mortality gradient. Although psychosocial stress has been experienced by working individuals throughout time, Kirby (2015) indicated the 1970s as a watershed for workplace stress in becoming the phenomenon, as it is understood today. That lower-class individuals experience more stresses and have a lower ability to cope with it, resulting in elevated mortality, is consistent with our findings.

It is more difficult to ascertain why the class gradient emerged earlier for women than for men. It is possible that complex interactions among early-life conditions, childbearing, and lifestyle contributed to the earlier emergence of class differences in adult mortality for women than for men. More research is clearly needed to clarify this issue.

## Data Availability

The data from SEDD is available subject to restrictions by the General Data Protection Regulation of the European Union (for personal information) through the Centre for Economic Demography, Lund University (https://www.ed.lu.se/databases/sedd/sedd-public-access). Linked data from Statistics Sweden are available after application (www.scb.se).

## Footnotes

1 For the period 1813–1968, we use a STATA program to create the episodes from IDS-type data (see Quaranta 2016). For the period after 1968, the episodes are created from the national registers.
